# Confinamento e Diagnóstico Cardiovascular em Época de Pandemia: O Difícil Equilíbrio no Fio da Navalha

**DOI:** 10.36660/abc.20220192

**Published:** 2022-04-07

**Authors:** Nuno Bettencourt

**Affiliations:** 1 Unidade de Investigação Cardiovascular Faculdade de Medicina do Porto Porto Portugal Unidade de Investigação Cardiovascular – Faculdade de Medicina do Porto, Porto – Portugal

**Keywords:** Doenças cardiovasculares/fisiopatologia, COVID-19, Pandemia, Determinação de Necessidades de Cuidados de Saúde/estatística e dados numéricos, Pesquisa/tendências, Cuidados Médicos/métodos, Disparidades nos Níveis de Saúde

A pandemia por COVID-19 teve um forte impacto na prestação de cuidados de saúde a nível mundial. As doenças cardiovasculares, incluindo as suas formas agudas não foram exceção. Logo nas primeiras semanas de pandemia, ainda no final do primeiro trimestre de 2020, foi notada uma franca redução dos recursos aos serviços de saúde quer em cuidados programados quer em admissões por síndromas coronárias agudas, com forte impacto no prognóstico imediato e com consequências futuras ainda não totalmente documentadas.^1,2^

Para além do impacto direto do vírus no miocárdio, que condiciona uma morbilidade e mortalidade não desprezível,^3-6^ também a redução do acesso a cuidados de saúde - que ocorreu tanto por uma canalização massiva dos recursos dos diferentes sistemas de saúde para o combate à pandemia, como pelo receio da população em recorrer a esses serviços durante a pandemia – está a ter um impacto importante no diagnóstico e tratamento da doença cardiovascular. Apesar da real dimensão deste impacto no prognóstico de pacientes com e sem COVID não estar ainda estabelecida é provável que a sua importância ultrapasse os efeitos diretamente determinados pela pandemia.^7^

Desde cedo, a comunidade cardiológica se apercebeu do impacto que quer a pandemia, de forma mais ou menos direta, quer as medidas usadas para a combater – nomeadamente os confinamentos obrigatórios e o distanciamento social – teriam no diagnóstico das doenças cardiovasculares.^8^

Um pouco por todo o mundo a comunidade científica organizou-se no sentido de promover um levantamento da nova realidade desencadeada pela pandemia de COVID-19 em termos de acesso aos cuidados de saúde na área cardiovascular. Para além de quantificar o seu real impacto na qualidade dos serviços prestados, este esforço coletivo tinha como objetivo adicional a identificação dos fatores que mais condicionaram esse acesso. Este conhecimento permite uma melhor compreensão do fenómeno e lança as bases para o desenvolvimento de estratégias que possam minimizar os seus efeitos quer em próximas vagas desta pandemia, quer em situações semelhantes que possam vir a ocorrer no futuro.

De entre os diferentes grupos que em poucas semanas se organizaram para conseguir concretizar este levantamento a nível mundial destacou-se a *International Atomic Energy Agency* que, partindo da sua rede internacional de investigação clínica pré-existente – INCAPS – alargou o seu âmbito e implantação, tendo conseguido uma participação verdadeiramente surpreendente. Com o envolvimento ativo de cardiologistas, radiologistas e de médicos de outras especialidades dedicados ao diagnóstico cardiovascular de todo o mundo, o consórcio INCAPS COVID conseguiu obter dados relativos aos volumes diagnósticos cardíacos no início da pandemia (março e abril de 2020), bem como os de março de 2019 – que funcionou como base de comparação representativa do período pré-pandémico. Estes dados foram integrados com os dados de distanciamento social a partir dos Relatórios de mobilidade da comunidade de Google e com os dados relativos à incidência de COVID-19 por país a partir de “Our World in Data”. Usando esta estratégia foi possível um levantamento da realidade a nível global e regional, permitindo avaliar os impactos sentidos nas diferentes modalidades diagnósticas em cardiologia, de acordo com a região,^8-11^ e, comparar estes dados entre diferentes áreas geográficas.^12^

Neste número da revista ABC, Cerci RJ et al.^13^ publicam os dados deste grupo relativo ao impacto da pandemia de COVID-19 na prestação de atendimento a doenças cardiovasculares na América Latina. Foram avaliados os volumes de diagnóstico cardíaco e determinada sua relação com a incidência de casos de COVID-19 e as medidas de distanciamento social usando dados de 194 centros de 19 países da América Latina.

Em comparação com o mês de março de 2019, os volumes dos procedimentos diagnósticos cardíacos diminuíram de forma acentuada (36% em março e 82% em abril de 2020), com pequenas variações entre as sub-regiões da América Latina. A queda foi mais relacionada ao distanciamento social do que ao aumento da incidência da COVID-19, verificando-se uma maior redução no número de procedimentos diagnósticos no mês de menor mobilidade (abril) – que coincidiu com os períodos mais rígidos de confinamento de cada país. Curiosamente esta queda percentual foi superior à verificada no mesmo período na Europa Ocidental (46% e 69%, respectivamente) e na América do Norte (39% e 68%, respectivamente), apesar de estas regiões estarem a vivenciar o primeiro pico de incidência, enquanto que na América Latina a pandemia estava apenas no seu início. Isto parece justificar-se pelo facto da maioria dos países da América Latina, terem introduzido o isolamento social logo em março de 2020, apesar do reduzido número de casos. Estes dados reforçam a importância de um melhor equilíbrio entre medidas de distanciamento social e acesso ao atendimento médico durante um surto pandêmico, uma vez que a redução do acesso aos cuidados de saúde tende a agravar as desigualdades pré-existentes na qualidade dos serviços prestados e pode ser particularmente relevante em regiões com elevada mortalidade cardiovascular.^14,15^
