# Supplementary material for: Confinamento e Diagnóstico Cardiovascular em Época de Pandemia: O Difícil Equilíbrio no Fio da Navalha
Source: Arq Bras Cardiol. 2022 Apr 7;118(4):754–5. [Article in Portuguese] doi: 10.36660/abc.20220192 (PMC9007008; doi:10.36660/abc.20220192)
Supplement: Translation

# Translation: Confinement and Cardiovascular Diagnosis in a Pandemic Season: The Difficult Balance on the Razor’s Edge

**Authors:** Nuno Bettencourt

**Affiliations:** 1 Portugal Unidade de Investigação Cardiovascular – Faculdade de Medicina do Porto, Porto – Portugal

**Keywords:** Cardiovascular Diseases/physiopathology, COVID-19, Pandemics, Needs Assessment/statistics & numerical data, Research/trends, Medical Care/methods, Health Status Disparities

The COVID-19 pandemic has had a strong impact on healthcare delivery worldwide. Cardiovascular diseases, including their acute forms, were no exception. In the first weeks of the pandemic, at the end of the first quarter of 2020, there was a clear reduction in resources to health services, both in scheduled care and in admissions for acute coronary syndromes, with a strong impact on the immediate prognosis and with future consequences not fully documented.^1,2^

In addition to the direct impact of the virus on the myocardium, which causes considerable morbidity and mortality,^3-6^ there is also a reduction in access to health care - which occurred both as a result of a massive channeling of resources from the different health systems to the fighting the pandemic, as well as the population’s fear of resorting to these services during the pandemic – is having an important impact on the diagnosis and treatment of cardiovascular disease. Although the real dimension of this impact on the prognosis of patients with and without COVID has not yet been established, its importance likely exceeds the effects directly determined by the pandemic.^7^

Early on, the cardiology community realized the impact that both the pandemic, more or less directly, and the measures used to combat it – namely mandatory confinements and social distancing – would have on the diagnosis of cardiovascular diseases.^8^

All over the world, the scientific community organized itself to promote a survey of the new reality triggered by the COVID-19 pandemic in terms of access to health care in the cardiovascular area. In addition to quantifying its real impact on the quality of services provided, this collective effort had the additional objective of identifying the most relevant factors conditioning health access. This knowledge allows a better understanding of the phenomenon and lays the foundations for developing strategies that can minimize its effects both in the next waves of this pandemic and in similar situations that may occur in the future.

Among the different groups that organized themselves in a few weeks to carry out this survey worldwide, the International Atomic Energy Agency stood out. Based on its pre-existing international network of clinical research – INCAPS – expanded its scope and implementation, having achieved a truly surprising turnout. With the active involvement of cardiologists, radiologists and physicians from other specialties dedicated to cardiovascular diagnosis from around the world, the INCAPS COVID consortium was able to obtain data regarding cardiac diagnostic volumes at the beginning of the pandemic (March and April 2020) as well as the March 2019 – which served as a representative comparison base for the pandemic loss. This data was integrated with social distancing data from Google’s Community Mobility Reports and data on COVID-19 incidence by country from “Our World in Data.” Using this strategy, it was possible to survey the reality at a global and regional level, allowing the assessment of the impacts felt in the different diagnostic modalities in cardiology, according to the region^8-11^ and, comparing these data between different geographic areas.^12^

In this issue of ABC journal, Cerci RJ et al.^13^ publish data from this group regarding the impact of the COVID-19 pandemic on the provision of care for cardiovascular diseases in Latin America. Cardiac diagnostic volumes were evaluated, and their relationship to COVID-19 case incidence and social distancing measures was determined using data from 194 centers in 19 Latin American countries.

Compared to March 2019, volumes of cardiac diagnostic procedures declined sharply (36% in March and 82% in April 2020), with small variations across Latin American subregions. The drop was more related to social distancing than to the increase in the incidence of COVID-19, with a greater reduction in the number of diagnostic procedures in the month of lower mobility (April) – which coincided with the strictest periods of confinement for each country. Interestingly, this percentage drop was higher than that seen in the same period in Western Europe (46% and 69%, respectively) and North America (39% and 68%, respectively). However, these regions were experiencing the first peak of incidence, while in Latin America, the pandemic just begining. This seems to be justified because most Latin American countries introduced social isolation as early as March 2020, despite the low number of cases. These data reinforce the importance of a better balance between social distancing measures and access to medical care during a pandemic outbreak. Reduced access to health care exacerbates pre-existing inequalities in the quality of services provided and can be particularly relevant in regions with high cardiovascular mortality.^14,15^
